# The efficacy and safety of the dipeptidyl peptidase-4 inhibitor saxagliptin in treatment-naïve patients with type 2 diabetes mellitus: a randomized controlled trial

**DOI:** 10.1186/1758-5996-4-36

**Published:** 2012-07-24

**Authors:** Robert Frederich, Robert McNeill, Niklas Berglind, Douglas Fleming, Roland Chen

**Affiliations:** 1 Bristol-Myers Squibb, Route 206 & Province Line Road, Princeton, NJ, 08543, USA; 2 PMG Research of Salisbury, 401 Mocksville Ave., Salisbury, NC, 28144, USA; 3 AstraZeneca R&D, Kärragatan 5, 431 83, Mölndal, Sweden

**Keywords:** DPP-4 inhibitor, Monotherapy, Saxagliptin, Titration, Type 2 diabetes mellitus

## Abstract

**Background:**

The aim of this study was to assess efficacy and safety of saxagliptin monotherapy for up to 76 weeks in patients with type 2 diabetes mellitus (T2DM) and inadequate glycemic control, with main efficacy assessment at 24 weeks.

**Methods:**

365 treatment-naïve patients with T2DM (HbA_1c_ 7.0%–10.0%) were treated with saxagliptin 2.5 mg q.A.M., saxagliptin 2.5 mg q.A.M. with possible titration to saxagliptin 5 mg, saxagliptin 5 mg q.A.M., saxagliptin 5 mg q.P.M., or placebo. After week 24, patients in all groups were eligible for titration to saxagliptin 10 mg based on HbA_1c_ ≥7%, and all unrescued placebo patients began blinded metformin 500 mg/day. Rescue with open-label metformin was available for patients with inadequate glycemic control.

**Results:**

At week 24, placebo-subtracted mean HbA_1c_ reduction from baseline (LOCF) was significantly greater in the saxagliptin treatment groups vs placebo, and remained greater through week 76. Serious adverse events (AEs) and discontinuations due to AEs were similar in saxagliptin and control groups; incidence of confirmed hypoglycemia was low across all treatment groups (saxagliptin-treated, 2 [0.7]; control, 1 [1.4]).

**Conclusions:**

In treatment-naïve patients with T2DM, saxagliptin monotherapy demonstrated statistically significant improvement in HbA_1c_ compared with placebo at 24 weeks and was generally well tolerated for up to 76 weeks.

**Trial registration:**

ClinicalTrials.gov Identifier: NCT00316082

## Background

After initiation of therapy, patients with type 2 diabetes mellitus (T2DM) are likely to require pharmacotherapeutic management for the remainder of their lives. Understanding the long-term efficacy and safety profiles of newer antidiabetic medications is necessary for optimizing treatment regimens over time. Incretin-based therapies, such as dipeptidyl peptidase-4 (DPP-4) inhibitors, are a class of recently developed antidiabetic agents for which long-term safety data are only now becoming available [1].

Saxagliptin is a potent, selective DPP-4 inhibitor approved as an adjunct to diet and exercise to improve glycemic control in adults with T2DM [2]. As monotherapy, saxagliptin 2.5, 5, and 10 mg led to statistically significant improvements in glycemic indices versus placebo at week 24, and was generally well tolerated in treatment-naïve patients with T2DM. Hypoglycemic events were comparable between saxagliptin treatment groups and placebo, and there were no increases in weight [3]. Currently, long-term efficacy and safety data for saxagliptin as add-on therapy to metformin [4], a sulfonylurea [5], or a thiazolidinedione [6], or as initial combination therapy with metformin [7], have been published, but long-term data have not been published for saxagliptin monotherapy at approved doses of 2.5 and 5 mg.

Here we report the saxagliptin experience at week 76 in a second treatment-naïve population. The current study was designed to demonstrate the efficacy and safety of saxagliptin monotherapy when used as a fixed-dose treatment and with titration in the initial treatment of hyperglycemia in patients with T2DM inadequately controlled with diet and exercise. The comparator to week 24 was placebo, converting thereafter to metformin 500 mg to week 76. This study also explored A.M. and P.M. dosing and early titration of saxagliptin 2.5 to 5 mg (approved doses) in the first 24 weeks. In order to evaluate whether uptitration from 5 mg to 10 mg provides any further improvement in glycemic control and to further characterize safety with the 10-mg dose, patients in all saxagliptin groups could be titrated to 10 mg in weeks 24 to 76 based on prespecified glycated hemoglobin (HbA_1c_) criteria. Data on time of day at which dosing occurs and titration are important to physicians, who must tailor treatment regimens for their patients.

## Methods

### Patients

Patients were recruited from 72 sites in the United States, Russia, India, and Taiwan. Inclusion and exclusion criteria were similar to those used and reported in the other saxagliptin monotherapy study [3]. Briefly, patients 18 to 77 years of age with T2DM and inadequate glycemic control (HbA_1c_ at screening 7.0% − 10.0%) with diet and exercise alone, body mass index (BMI) ≤40 kg/m^2^, and C-peptide ≥1.0 ng/mL were eligible. All patients were treatment naïve, defined as those who had never received medical treatment for diabetes (insulin and/or oral antihyperglycemic agents) or had received medical treatment for diabetes for a total of <6 months since original diagnosis. Exclusion criteria included symptoms of poorly controlled diabetes; history of diabetic ketoacidosis or hyperosmolar non-ketotic coma; insulin therapy within 1 year of screening; cardiovascular event within 6 months prior to study entry or New York Heart Association stage III/IV congestive heart failure (CHF) and/or known left ventricular ejection fraction ≤40%; significant renal history, alcohol or drug abuse within the previous year; treatment with potent CYP3A4 inhibitors or inducers; immunocompromised individuals; active liver disease or clinically significant abnormal results on hepatic, renal, endocrine, metabolic, or hematologic screening tests.

This study was performed in accordance with Good Clinical Practice as defined by the International Conference on Harmonisation and the ethical principles set forth in the Declaration of Helsinki. Patients freely provided written informed consent. The study protocol, amendments, and patient informed consent were approved by the institutional review board/independent ethics committee for each participating site prior to study initiation.

### Study design

This was a 24-week, phase 3, randomized, 5-arm, parallel-group, double-blind, placebo-controlled, multicenter trial with an extension to 76 weeks. The 24-week short-term period was designed to assess the efficacy and safety of saxagliptin monotherapy as 2.5 mg q.A.M. without titration, 2.5 mg q.A.M. with titration, 5 mg q.A.M., and 5 mg q.P.M. The 52-week extension included patients who either completed the short-term, double-blind period or were rescued due to lack of glycemic control. Rescue medication was also available in the long-term extension based on predefined criteria. The long-term extension was designed to assess long-term safety and glycemic parameters for each saxagliptin regimen. Eligible patients were instructed on diet and exercise in accordance with the American Diabetes Association (ADA) or corresponding guidelines in each country. Following completion of the lead-in period, patients were randomized (1:1:1:1:1) to saxagliptin 2.5 mg q.A.M., saxagliptin 2.5 mg with possible titration to 5 mg q.A.M. (2.5/5 mg q.A.M.), saxagliptin 5 mg q.A.M., saxagliptin 5 mg q.P.M., or placebo. In patients randomized to saxagliptin 2.5/5 mg q.A.M., saxagliptin was initiated at 2.5 mg and titrated to 5 mg if mean fasting plasma glucose (FPG) was ≥150 mg/dL at week 4, ≥140 and ≤220 mg/dL at week 8, or ≥126 and ≤200 mg/dL at weeks 12 and 24.

Patients with inadequate glycemic control during the short-term period were eligible for add-on, open-label metformin rescue therapy dosed beginning with 500 mg/day and titrated as tolerated to a maximum of 2000 mg/day. Rescue criteria for lack of glycemic control based upon FPG during the short-term period became progressively more strict at week 6 (>240 mg/dL); week 8 (>220 mg/dL); and weeks 12, 16, 20, and 24 (>200 mg/dL). Patients rescued from the short-term period were reported as early discontinuations for lack of efficacy prior to entering the long-term extension.

Patients who completed or were rescued during the short-term period were eligible to enter the 52-week long-term extension. During the long-term extension, patients who had completed all visits and had not met hyperglycemia rescue criteria in the short-term period were allowed to titrate up to a maximum saxagliptin dose of 10 mg if HbA_1c_ was >8.0% at weeks 30, 37, and 50 or >7.5% at week 63. Patients who had received placebo in the short-term period and were not rescued were switched to blinded metformin 500 mg in the long-term extension. Titration of blinded metformin was prohibited. Patients who met hyperglycemia rescue criteria during the short-term period remained on the same randomized dose and treatment assigned throughout the long-term extension, but received open-label metformin, which could be titrated to a maximum of 2000 mg/day, in addition to their study medication. Patients with lack of glycemic control during the long-term treatment period (HbA_1c_ >8.0% at weeks 30, 37, and 50 or >7.5% at week 63) were also eligible for rescue with open-label metformin 500 mg titrated as tolerated to a maximum of 2000 mg/day. Patients in the comparator group were defined as the “placebo group” up to week 24 and as the “control group” once they entered the long-term extension.

### Study endpoints and assessments

The primary efficacy endpoint was the change from baseline HbA_1c_ at week 24 for the saxagliptin 2.5 mg q.A.M., 2.5/5 mg q.A.M., 5 mg q.A.M., and placebo groups. Secondary endpoints for all treatment groups in the short-term period were change from baseline FPG, the proportion of patients achieving HbA_1c_ <7.0%, and change from baseline in area under the curve (AUC) from 0 to 180 minutes for postprandial glucose (PPG-AUC) response to an oral glucose tolerance test (OGTT), and for the saxagliptin 5 mg q.P.M. group, change from baseline HbA_1c_ at week 24.

Glycemic parameters evaluated for each saxagliptin treatment group in the 52-week long-term extension included time to rescue for failing to achieve prespecified glycemic targets or discontinuations due to lack of efficacy; change from baseline HbA_1c_, FPG, and PPG-AUC over time; and change in HbA_1c_ from first titration in long-term extension to 13 weeks post–first titration.

Safety and tolerability assessments included adverse events (AEs), serious AEs (SAEs), related AEs, discontinuations from study medication due to AEs, and deaths. Laboratory values, electrocardiograms, vital signs, physical examinations, and body weight were also assessed.

### Data analysis

For the short-term period, a primary efficacy analysis was performed for the endpoint of change in HbA_1c_ from baseline to week 24 comparing the saxagliptin q.A.M. treatment groups to placebo, with missing data imputed on a last-observation-carried-forward (LOCF) basis. A two-step, parallel gatekeeping methodology was used to preserve the overall type I error rate at the 0.05 level [8]. As a first step, comparisons were made between the saxagliptin 2.5 mg q.A.M. and 5 mg q.A.M. treatment groups versus the placebo group. If either saxagliptin group showed significance for HbA_1c_ at the α = 0.027 level versus placebo, the second step comparison would use the same α level; if both first-step comparisons reached significance, α = 0.05 would be used. The second comparison was the saxagliptin 2.5/5 mg q.A.M. titrated group versus placebo. Assuming a standard deviation (SD) of 1.1%, 62 patients per treatment group would provide 90% power to detect a difference in means of 0.7% between saxagliptin 2.5 mg q.A.M. or 5 mg q.A.M versus placebo.

The statistical testing of the secondary efficacy endpoints proceeded in a sequential manner, to control the type I error rate within each treatment group at the 0.05 level. The first secondary endpoint was the change in HbA_1c_ from baseline to week 24 in the saxagliptin 5 mg q.P.M. treatment group. For all treatment groups, secondary endpoints were the change in FPG from baseline to week 24, the percentage of patients achieving HbA_1c_ <7.0% at week 24, and the change from baseline to week 24 in AUC from 0 to 180 minutes for PPG response to an OGTT. Mean changes from baseline to week 24 (LOCF) were compared between each investigative treatment group and the placebo group based on adjusted means generated using an analysis of covariance (ANCOVA) model. Within this framework, point estimates and 95% confidence intervals (CIs) were calculated for the mean changes from baseline within each treatment group, as well as for the difference in mean change from baseline between each investigative treatment group and the placebo group. The percentages of patients achieving HbA_1c_ <7.0% at week 24 (LOCF) were compared between each investigative treatment group and the placebo group using the Fisher exact test.

Patients in the placebo group began receiving active therapy with metformin as they entered the long-term extension. Changes from baseline HbA_1c_ during the whole study period were reported. For these longer-term analyses, a repeated-measures analysis was performed, as it more appropriately addresses missing data compared with the LOCF analysis in the long-term extension, in which fewer data are available for analysis due to rescue or study discontinuation.

A total of 50 patients (14% of the randomized patients) had OGTT samples that were rendered invalid when incorrect quantities of glucose were administered. Patients were to begin administration of 75 g of oral glucose solution at time 0 minutes during the OGTT. However, bottles containing more than 75 g of oral glucose solution were shipped and administered at certain study sites in Russia. At those sites, patients who were dispensed an amount in excess of the prespecified 75 g of oral glucose solution were not included in any analysis of OGTT parameters.

Safety analyses were performed using all data in the short-term period and long-term extension. AEs were tabulated and classified according to the Medical Dictionary for Regulatory Activities (MedDRA) version 11.1 at the preferred term level and grouped by system organ class (SOC). Hypoglycemia was evaluated separately from other AEs; confirmed hypoglycemia was defined by a fingerstick glucose value ≤50 mg/dL in the presence of associated symptoms.

## Results

### Patient disposition

**Figure 1:**
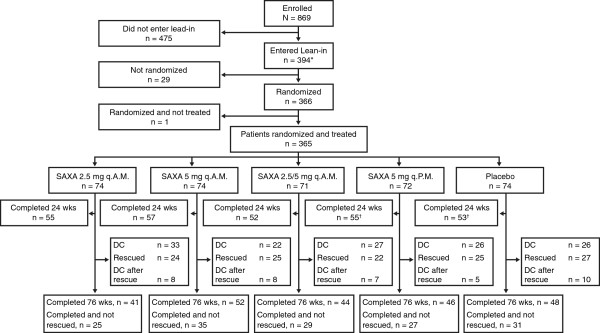
**Patient disposition short-term period (week 24) and long-term extension (week 76).** Rescued patients include all patients who were rescued in short-term period or long-term extension period. Rescued patients may later have discontinued from the study.*One patient did not enter lead-in period but was directly randomized to double-blind treatment. ^†^One patient in the saxagliptin 5 mg q.P.M. group and one patient in the placebo group completed week 24, but discontinued treatment (lost to follow-up and poor compliance, respectively).

Patient disposition for the 24-week, short-term period and long-term extension is illustrated in Figure 1. A total of 869 patients completed the informed consent covering all phases of the study and were screened; 394 entered the lead-in period. Following a 2-week, single-blind, dietary and exercise placebo lead-in period, 365 patients were randomized (74, 74, 71, 72, and 74 patients in the saxagliptin 2.5 mg q.A.M., 5 mg q.A.M., 2.5/5 mg q.A.M., and 5 mg q.P.M., and placebo groups, respectively) and treated with double-blind therapy; 272 patients completed 24 weeks of short-term treatment without rescue. Of the 311 patients who entered the long-term extension (including 266 of the 272 short-term non-rescued completers), 231 completed the study (76 weeks), 147 without being rescued. Total completion and completion without rescue proportions were highest in the saxagliptin 5 mg q.A.M. group, and lowest in the 2.5 mg q.A.M. group, with the 3 remaining groups comparable and intermediate. Completion *without titration* during the long-term extension from the randomized dose was numerically higher in the groups randomized to saxagliptin 5 mg (17.9%–26.4%) than in those randomized to saxagliptin 2.5 mg (9.3%–13.7%). In consequence, by the end of the study even in the groups in which the initial randomized dose was 2.5 mg (saxagliptin 2.5 mg q.A.M. and 2.5/5 mg q.A.M.), nearly all of the patients were taking saxagliptin 5 or 10 mg.

Among patients who completed the short-term period, 42.6%, 82.1%, 68.6%, and 73.6% of patients were eventually titrated to 10 mg in the saxagliptin 2.5 mg q.A.M., 5 mg q.A.M., 2.5/5 mg q.A.M., and 5 mg q.P.M. groups, respectively. Most of the patients in the saxagliptin 2.5/5 mg q.A.M. group had already been titrated to 5 mg by week 24.

Mean duration of exposure ranged from 54.6 weeks (2.5 mg q.A.M.) to 62.2 weeks (5 mg q.A.M.) across the saxagliptin treatment groups and was 58.8 weeks in the control group. The median duration of exposure to study medication was approximately 75 weeks across all treatment groups.

### Patient demographics

Patient demographic characteristics were generally balanced across the randomized treatment groups. However, some differences were observed in gender, body weight, BMI, and HbA_1c_ (Table 1). The proportion of men was highest in the saxagliptin 2.5/5 mg q.A.M. group (52.1%) and lowest in the saxagliptin 2.5 mg q.A.M. group (33.8%). The mean body weight of patients receiving placebo was 85.4 kg, and ranged from 83.3 to 86.5 kg among patients receiving saxagliptin. Median baseline HbA_1c_ for all of the saxagliptin treatment groups was 7.8% or 7.9% whereas the median HbA_1c_ for the placebo group was 7.6%. Overall 70% of patients were white and 23% were Asian. The mean duration of diabetes was 1.7 years, and mean baseline HbA_1c_ was 7.9% for the entire study population. This unselected patient population demonstrated a substantial prevalence of risk factors for cardiovascular (CV) events at baseline. Nine percent had prior CHF history, 13% had coronary artery disease history, and 5% had prior myocardial infarction. A majority of patients had a history of hypertension (58%), and a substantial minority had a history of obesity (46%), hypercholesterolemia (33%), mixed dyslipidemia (18%), and tobacco use (25%).

**Table 1:** Baseline demographic and clinical characteristics

**Characteristic**	**SAXA 2.5 mg q.A.M. (n = 74)**	**SAXA 5 mg q.A.M. (n = 74)**	**SAXA 2.5/5 mg q.A.M. (n = 71)**	**SAXA 5 mg q.P.M. (n = 72)**	**Placebo (n = 74)**
Age, years, mean (SD)	55.2 (10.44)	54.7 (9.71)	54.3 (10.93)	55.1 (10.35)	55.6 (10.32)
Age ≥65 years, n (%)	16 (21.6)	12 (16.2)	12 (16.9)	11 (15.3)	13 (17.6)
Gender, n (%)					
Male	25 (33.8)	38 (51.4)	37 (52.1)	33 (45.8)	35 (47.3)
Female	49 (66.2)	36 (48.6)	34 (47.9)	39 (54.2)	39 (52.7)
Race, n (%)					
White	50 (67.6)	49 (66.2)	54 (76.1)	48 (66.7)	53 (71.6)
Black/African American	5 (6.8)	5 (6.8)	2 (2.8)	8 (11.1)	4 (5.4)
Asian	18 (24.3)	20 (27)	14 (19.7)	16 (22.2)	17 (23)
Other	1 (1.4)	0	1 (1.4)	0	0
Weight, kg, mean (SD)	83.8 (16.70)	86.5 (20.71)	85.4 (17.25)	83.3 (19.10)	85.4 (14.40)
BMI, kg/m^2^, mean (SD)	30.4 (4.84)	31.0 (5.23)	30.6 (4.72)	29.6 (5.37)	31.1 (4.54)
Duration of diabetes, years, mean (SD)	1.2 (1.6)	1.7 (2.4)	2.0 (2.9)	2.0 (5.2)	1.7 (2.8)
HbA_1c_, %, mean (SD)	8.0 (0.8)	8.0 (0.9)	8.0 (1.1)	7.9 (0.9)	7.8 (1.0)
<8.0%, n (%)	40 (54.1)	40 (54.1)	37 (52.1)	38 (52.8)	47 (63.5)
≥8.0% to < 9.0%, n (%)	23 (31.1)	27 (36.5)	18 (25.4)	24 (33.3)	15 (20.3)
≥9.0%, n (%)	11 (14.9)	7 (9.5)	16 (22.5)	10 (13.9)	12 (16.2)
FPG, mg/dL, mean (SD)	158 (33.0)	162 (35.6)	171 (51.8)	160 (44.6)	160 (46.2)
Hypertension, n (%)	40 (54.1)	36 (48.7)	48 (67.6)	41 (56.9)	47 (63.5)
Hypercholesterolemia, n (%)	25 (33.8)	22 (29.7)	32 (45.1)	23 (31.9)	17 (23.0)
Mixed dyslipidemia, n (%)	13 (17.6)	14 (18.9)	15 (21.1)	13 (18.1)	9 (12.2)
CHF, n (%)	6 (8.1)	8 (10.8)	7 (9.9)	4 (5.6)	7 (9.5)
Coronary artery disease, n (%)	9 (12.2)	8 (10.8)	15 (21.1)	8 (11.1)	9 (12.2)
Prior MI, n (%)	4 (5.4)	2 (2.7)	5 (7.0)	4 (5.6)	4 (5.4)

BMI: body mass index; CHF: congestive heart failure; FPG: fasting plasma glucose; HbA_1c_: glycated hemoglobin; MI: myocardial infarction; SAXA: saxagliptin; SD: standard deviation.

### Change in glycemic parameters

#### Short-term period (week 24)

**Figure 2:**
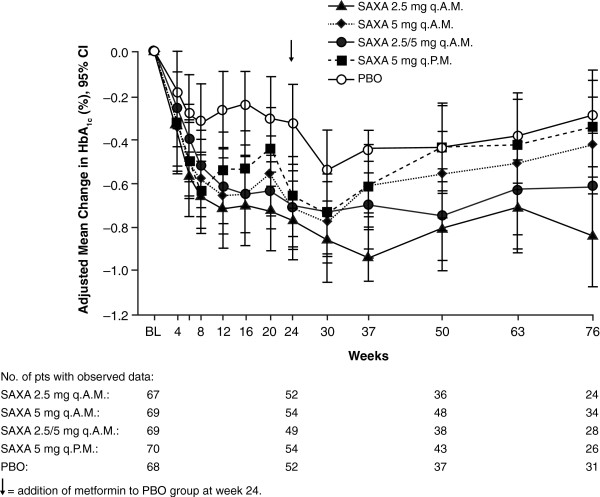
**Adjusted mean changes from baseline HbA**_**1c**_** over 76 weeks (repeated-measures analysis).** Randomized and treated patients. Patients who received placebo in the short-term period were switched to blinded metformin 500 mg at week 24. Titration of blinded metformin was not allowed. BL: baseline; HbA_1c_: glycated hemoglobin; PBO: placebo; SAXA: saxagliptin.

The mean adjusted changes from baseline HbA_1c_ and FPG, the percentage of patients achieving HbA_1c_ <7%, and mean adjusted changes from baseline PPG-AUC during the short-term period are presented in Table 2. There were reductions from baseline HbA_1c_ (LOCF) in all saxagliptin treatment groups (−0.61% to −0.71%) and a smaller reduction in the placebo group (−0.26%) (Figure 2). All reductions with saxagliptin were significantly greater than with placebo (*P* = 0.002, *P* = 0.006, *P* = 0.012, and *P* = 0.016 for saxagliptin 2.5 mg q.A.M., 5 mg q.A.M., 2.5/5 mg q.A.M., and 5 mg q.P.M., respectively). Reductions from baseline FPG (LOCF) of −10.7 to −12.5 mg/dL were observed in the saxagliptin morning dosing groups. All were significantly greater than with placebo (*P* = 0.020, *P* = 0.027, and *P* = 0.013 for saxagliptin 2.5 mg q.A.M., 5 mg q.A.M., and 2.5/5 mg q.A.M., respectively). The reduction in FPG in the saxagliptin 5 mg q.P.M. treatment group, −7.9 mg/dL, did not reach significance relative to placebo (*P* = 0.076). Although the proportions of patients achieving an HbA_1c_ <7% were slightly larger for all saxagliptin treatment groups relative to placebo, the differences were not statistically significant. Consistent mean reductions from baseline in PPG-AUC were observed for all saxagliptin groups compared with placebo. However, the placement of this endpoint in the sequential testing procedure prohibited interpretation of statistical significance.

**Table 2:** Changes in glycemic parameters from baseline to week 24 (LOCF)

	**SAXA 2.5 mg q.A.M. (n = 74)**	**SAXA 5 mg q.A.M. (n = 74)**	**SAXA 2.5/5 mg q.A.M. (n = 71)**	**SAXA 5 mg q.P.M. (n = 72)**	**Placebo (n = 74)**
**HbA**_**1c**_** (%)**					
n	67	69	69	70	68
BL, mean (SE)	8.0 (0.11)	7.9 (0.11)	8.0 (0.13)	7.9 (0.11)	7.8 (0.11)
Week 24 mean (SE)	7.3 (0.11)	7.3 (0.13)	7.4 (0.14)	7.3 (0.12)	7.6 (0.14)
Adj change from BL, mean (SE)	−0.71 (0.103)	−0.66 (0.102)	−0.63 (0.102)	−0.61 (0.101)	−0.26 (0.103)
Difference from placebo (SE)	−0.45 (0.146)	−0.40 (0.145)	−0.37 (0.145)	−0.35 (0.144)	
*P*-value vs placebo	0.002*	0.006*	0.012*	0.016*	
**FPG (mg/dL)**					
n	70	71	71	71	71
BL, mean (SE)	157 (4.0)	162 (4.2)	171 (6.2)	160 (5.3)	159 (5.4)
Week 24 mean (SE)	147 (3.8)	151 (5.9)	155 (5.9)	152 (5.9)	163 (6.3)
Adj change from BL, mean (SE)	−11 (4.5)	−11 (4.5)	−13 (4.5)	−8 (4.5)	3 (4.5)
Difference from placebo (SE)	−15 (6.3)	−14 (6.3)	−16 (6.3)	−11 (6.3)	
*P*-value vs placebo	0.020*	0.027*	0.013*	0.076	
**Patients achieving HbA**_**1c**_** <7% (%)**	35.8	44.9	43.5	38.6	35.3
Difference from placebo (%)	0.5	9.6	8.2	3.3	
*P*-value vs placebo	1.0000	0.2968	0.3832	^†^	
**PPG–AUC (mg·min/dL)**					
n	48	48	47	43	47
BL, mean (SE)	47432 (1496.6)	50417 (1561.5)	50032 (1684.7)	47078 (1941.9)	47640 (1759.7)
Week 24 mean (SE)	39798 (1347.0)	41562 (1489.3)	41745 (1739.2)	41530 (1962.7)	44861 (1854.7)
Adj change from BL, mean (SE)	−8014 (1246.9)	−8218 (1249.1)	−7781 (1261.0)	−6048 (1318.2)	−3088 (1259.7)
Difference from placebo (SE)	−4927 (1771.1)	−5130 (1776.4)	−4694 (1784.3)	−2961 (1821.5)	
*P*-value vs placebo	^†^	^†^	^†^	^†^	

Randomized patients data set.

*Statistically significant at prespecified level.

^†^*P*-values only presented when permitted by sequential testing procedure.

Adj: adjusted; BL: baseline; FPG: fasting plasma glucose; HbA_1c_: glycated hemoglobin; PPG-AUC: postprandial glucose–area under the curve; SAXA: saxagliptin; SE: standard error.

### Body weight

Overall, there were reductions in mean body weight in all treatment groups. The mean (SE) change from baseline body weight (LOCF) was −0.3 kg (0.35), –0.9 kg (0.31), –0.9 kg (0.41), –0.4 kg (0.36), and −1.3 kg (0.40) for saxagliptin 2.5 mg q.A.M., 5 mg q.A.M., 2.5/5 mg q.A.M., 5 mg q.P.M., and placebo treatment groups, respectively.

#### Long-term extension (week 76)

Upon entry to the long-term extension, all patients in the placebo group who did not receive rescue therapy were placed on blinded metformin for the duration of the study.

By 13 weeks post-titration, most patients in the saxagliptin 5 mg q.A.M., 2.5/5 mg q.A.M., and 5 mg q.P.M. treatment groups had been titrated to saxagliptin 10 mg, while most of the patients in the saxagliptin 2.5 mg q.A.M. treatment group had been titrated to saxagliptin 5 mg. Mean changes in HbA_1c_ from time of first titration in the long-term extension to 13 weeks after titration were small and inconsistent in the saxagliptin treatment groups (Additional file 1). Titration from saxagliptin 2.5 to 5 mg q.A.M. gave a small further reduction in mean (SE) HbA_1c_ (−0.12% [0.072]) and titration in the other treatment groups (where nearly all patients were at 5 mg at the start of the long-term extension and titration was from 5 to 10 mg) showed a small worsening in mean (SE) HbA_1c_ over the 13-week timeframe (0.09% [0.105], 0.11% [0.086], and 0.02% [0.120] for saxagliptin 5 mg q.A.M., 2.5/5 mg q.A.M., and 5 mg q.P.M., respectively).

Mean adjusted changes in glycemic parameters from baseline to week 76 (repeated-measures analysis) are shown in Table 3 and Figure 2. The reduction from baseline HbA_1c_ persisted at week 76 (−0.34% to −0.84%) but was attenuated to a variable degree in the groups initially treated with saxagliptin 5 mg q.A.M, 2.5/5 mg q.A.M., and 5 mg q.P.M. but showed no attenuation in the group initially treated with saxagliptin 2.5 mg q.A.M. At week 24 the reduction from baseline HbA_1c_ in the placebo group (repeated-measures analysis) was −0.33%. In the control group, which received blinded metformin 500 mg/day from week 24, mean change from baseline in HbA_1c_ at week 76 was −0.29% (repeated-measures analysis).

**Table 3:** Changes in glycemic parameters from baseline to week 76

	**SAXA 2.5 mg q.A.M. (n = 74)**	**SAXA 5 mg q.A.M. (n = 74)**	**SAXA 2.5/5 mg q.A.M. (n = 71)**	**SAXA 5 mg q.P.M. (n = 72)**	**Control (n = 74)**
**HbA**_**1c**_** (%) (repeated measures analysis)**					
BL, n	67	69	69	70	68
n with observed data at 76 weeks	24	34	28	26	31
BL, mean (SE)	8.0 (0.11)	7.9 (0.11)	8.0 (0.13)	7.9 (0.11)	7.8 (0.11)
Adj mean change from BL, mean (SE)	−0.84 (0.122)	−0.41 (0.108)	−0.60 (0.118)	−0.34 (0.117)	−0.29 (0.114)
95% CI	(−1.07, –0.60)	(−0.63, –0.20)	(−0.83, –0.37)	(−0.57, –0.12)	(−0.52, –0.07)
FPG (mg/dL) (repeated measures analysis)*					
BL, n	70	71	71	71	72
n with observed data at 76 weeks	20	28	22	23	26
BL, mean (SE)	157 (4.0)	162 (4.2)	171 (6.2)	160 (5.3)	160 (5.5)
Adj mean change from BL, mean (SE)	−12 (5.0)	−1 (4.4)	−15 (4.9)	1 (4.8)	0.1 (4.6)
95% CI	(−21.8, –2.1)	(−10.1, 7.3)	(−24.0, –4.9)	(−8.4, 10.3)	(−9.0, 9.1)
**HbA**_**1c**_** <7.0% (LOCF)**					
N	67	69	69	70	68
n (%)	27 (40.3)	22 (31.9)	30 (43.5)	22 (31.4)	23 (33.8)
95% CI	(28.5, 53.0)	(21.2, 44.2)	(31.6, 56.0)	(20.9, 43.6)	(22.8, 46.3)
PPG–AUC (mg·min/dL) (repeated measures analysis)*					
BL, n	44	43	41	39	41
n with observed data at 76 weeks	30	35	27	29	33
BL, mean (SE)	47053 (1529.8)	49512 (1621.9)	48084 (1673.4)	44498 (1813.4)	46111 (1938.3)
Adj mean change from BL, mean (SE)	−5859 (1498.3)	−4163 (1429.2)	−8511 (1571.7)	−4700 (1547.4)	−3788 (1465.6)
95% CI	(−8821, –2897)	(−6988, –1338)	(−11618, –5404)	(−7759, –1642)	(−6685, –891)

*Value rounded to nearest whole number

Adj: adjusted; BL: baseline; FPG: fasting plasma glucose; HbA_1c_: glycated hemoglobin; LOCF: last observation carried forward; PPG–AUC: postprandial glucose-area under the curve; SAXA: saxagliptin; SE: standard error

The mean adjusted change from baseline FPG (repeated-measures analysis) was sustained up to week 76 in the saxagliptin groups originally treated with 2.5 mg q.A.M. and 2.5/5 mg q.A.M., but not in the saxagliptin 5 mg q.A.M. and 5 mg q.P.M. groups (Table 3). The control group, to which metformin had been added after week 24, consistently showed no change from baseline FPG throughout the study. The proportion of patients achieving HbA_1c_ <7% at week 76 was similar to that at week 24 (Tables 2 and 3). Consistently numerically greater mean reductions from baseline PPG-AUC were observed for all saxagliptin groups compared with the control at week 76 (Table 3).

The percentage of patients who discontinued for lack of glycemic control or required rescue for meeting prespecified glycemic criteria at week 24 was higher in the placebo group (16.2%) than in the saxagliptin groups (12.2%, 13.5%, 14.1%, and 11.1% for the saxagliptin 2.5 mg q.A.M., 5 mg q.A.M., 2.5/5 mg q.A.M., and 5 mg q.P.M., respectively). At week 76, the percentages of patients who discontinued for lack of glycemic control or required rescue were 37.8% in the control group and 33.8%, 33.8%, 32.4%, and 36.1% in the saxagliptin 2.5 mg q.A.M., 5 mg q.A.M., 2.5/5 mg q.A.M., and 5 mg q.P.M. groups, respectively.

For all treatment groups in the short-term period and long-term extension, there were no clinically meaningful changes from baseline in either systolic or diastolic blood pressure. Similarly, there were no clinically relevant changes in fasting lipid parameters in any treatment group. With the exception of patients in the saxagliptin 5 mg q.P.M. group (+0.1 kg), a small reduction from baseline body weight was maintained in all treatment groups (−0.5 to −1.0 kg).

### Safety and tolerability

Overall, saxagliptin was generally well tolerated in the short-term period and long-term extension (Table 4). The proportion of patients experiencing ≥1 AE was numerically greater in those receiving saxagliptin (71.1%) versus control (55.4%). During the short-term + long-term period, at the SOC level, the proportion of patients with AEs was similar in the saxagliptin-treated patients and patients in the control group, except in the following SOCs: infections and infestations (41.9% vs 24.3%); gastrointestinal disorders (23.7% vs 10.8%); injury, poisoning and procedural complications (16.8% vs 6.8%); and general disorders and administration site conditions (15.1% vs 9.5%). For each of these SOCs, there were no single preferred terms or closely related terms that fully accounted for these findings. Saxagliptin was not associated with the development of opportunistic infections. The most common AEs by preferred term occurring ≥5% in all saxagliptin treated patients are listed in Table 4. AEs occurring ≥5% in any treatment group by preferred term are listed in Additional file 2.

**Table 4:** Adverse event summary: short-term period and long-term extension

	**SAXA 2.5 mg q.A.M. (n = 74)**	**SAXA 5 mg q.A.M. (n = 74)**	**SAXA 2.5/5 mg q.A.M. (n = 71)**	**SAXA 5 mg q.P.M. (n = 72)**	**All SAXA (n = 291)**	**Control (n = 74)**
Adverse events n (%)^*^						
≥1 AE	49 (66.2)	54 (73.0)	53 (74.6)	51 (70.8)	207 (71.1)	41 (55.4)
≥1 related AE	17 (23.0)	17 (23.0)	14 (19.7)	10 (13.9)	58 (19.9)	11 (14.9)
Deaths	0	0	2 (2.8)	0	2 (0.7)	0
≥1 SAE	7 (9.5)	8 (10.8)	7 (9.9)	4 (5.6)	26 (8.9)	5 (6.8)
≥1 related SAE	1 (1.4)	0	0	0	1 (0.3)	1 (1.4)
Discontinuation due to AE	4 (5.4)	2 (2.7)	5 (7.0)	1 (1.4)	12 (4.1)	3 (4.1)
Discontinuation due to SAEs	1 (1.4)	0	2 (2.8)	0	3 (1.0)	2 (2.7)
Adverse events (≥5% All SAXA)^†^						
URTI	11 (14.9)	10 (13.5)	11 (15.5)	11 (15.3)	43 (14.8)	7 (9.5)
Nasopharyngitis	3 (4.1)	4 (5.4)	3 (4.2)	5 (6.9)	15 (5.2)	3 (4.1)
Diarrhea	7 (9.5)	4 (5.4)	1 (1.4)	4 (5.6)	16 (5.5)	1 (1.4)
Pain in extremity	2 (2.7)	3 (4.1)	5 (7.0)	5 (6.9)	15 (5.2)	1 (1.4)
Reported hypoglycemia^‡^	3 (4.1)	6 (8.1)	6 (8.5)	6 (8.3)	21 (7.2)	3 (4.1)
Confirmed hypoglycemia^**^	0	1 (1.4)	0	1 (1.4)	2 (0.7)	1 (1.4)
Exposure, weeks, mean (SD)^***^	54.6 (27.6)	62.2 (24.3)	56.8 (27.2)	59.8 (25.0)	−	58.8 (25.8)

^*^Includes hypoglycemia

^†^Excludes hypoglycemia

^‡^Reported hypoglycemia was defined as events consistent with signs or symptoms of hypoglycemia with or without documented blood glucose levels

^**^Confirmed hypoglycemia defined as fingerstick glucose value ≤50 mg/dL in the presence of associated symptoms

^***^Exposure defined as the time from the first day to the last day, inclusive, that a patient took study medication. Exposure data includes rescue

AE: adverse event; SAE: serious adverse event; SAXA: saxagliptin; SD: standard deviation; URTI: upper respiratory tract infection

The incidence of SAEs was generally similar across treatment groups throughout the study: 8.9% of saxagliptin-treated patients and 6.8% of control patients. For saxagliptin, the most common SAE SOC was injury, poisoning and procedural complications (2.1% saxagliptin, 0% control): 3 of these 6 events were protocol-mandated SAEs of “overdose.” Two patients in the saxagliptin group were reported to have an AE of accidental overdose of what was determined to be placebo (looking like the metformin taken by control patients) and the third patient took 5 extra saxagliptin 2.5 mg pills over 14 days. All patients were completely asymptomatic. For the control group, the most common SAE SOC was “cardiac” (1.7% saxagliptin, 2.7% control). The SAEs in this SOC for the saxagliptin treatment groups included 1 event each of angina pectoris, unstable angina, atrial fibrillation, acute cor pulmonale, and coronary artery disease, and 2 events with the preferred term of “acute myocardial infarction” in the control group. The only SAE in the neurological SOC was a transient ischemic attack in the control group. Three SAEs in 2 patients were reported as related to the study drug: 1 patient in the saxagliptin 2.5 mg q.A.M. group presented with cellulitis and intertrigo, and 1 patient in the control group who had been previously rescued with metformin in the long-term extension presented with severe hypoglycemia which resolved the same day without treatment.

A total of 12 (4.1%) patients treated with saxagliptin discontinued due to AEs; 3 (1.0%) of these patients discontinued due to SAEs. Similarly, 3 (4.1%) patients in the control group discontinued due to AEs; 2 (2.7%) patients discontinued due to SAEs. Two deaths occurred in the saxagliptin 2.5/5 mg q.A.M. group, neither of which was considered by study investigators to be treatment related. One patient with a history of splenectomy due to a bull-riding accident died on day 54 due to pneumococcal sepsis. The second patient, who had elevated alkaline phosphatase at study entry, discontinued the study on day 13 due to pancreatic carcinoma and later died on day 502 due to pancreatic carcinoma and metastatic liver cancer.

The proportion of patients with hypoglycemic AEs was 4.1%, 8.1%, 8.5%, and 8.3% in the saxagliptin 2.5 mg q.A.M., 5 mg q.A.M., 2.5/5 mg q.A.M., and 5 mg q.P.M. treatment groups, respectively, and 4.1% in the control group. There were 2 cases of confirmed (defined as fingerstick glucose ≤50 mg/dL with associated symptoms) mild hypoglycemia in saxagliptin-treated patients, and 1 case of confirmed hypoglycemia in the control group receiving metformin (this was not the patient in the control group noted previously with the hypoglycemia SAE).

Prespecified “acute cardiovascular AEs” [9], which include myocardial infarction and other patient important CV events, were reported in 2 patients treated with saxagliptin (0.7%) and 3 with control (4.1%). The overall incidences of marked laboratory abnormalities were similar in all treatment groups. Other laboratory parameters, including hematologic, hepatic, and renal safety tests, showed no drug-related signal (data not shown). There were no clinically relevant changes in electrocardiograms or vital signs (data not shown).

## Discussion

In treatment-naïve patients with T2DM, once-daily saxagliptin lowered blood glucose concentrations relative to baseline after 24 weeks and was generally well tolerated through week 76 in all treatment groups. These findings were largely similar to those reached in the previously published saxagliptin monotherapy trial [3]. However, it is noteworthy that this study also explored the effects of dose titration and P.M. versus A.M. dosing, and that the patient populations differed with respect to important baseline characteristics (including baseline CV risk, HbA_1c_, and FPG).

At week 24, adjusted mean changes from baseline HbA_1c_ demonstrated in the 4 saxagliptin treatment groups (−0.61% to −0.71%) were comparable and consistent. The adjusted mean change from baseline HbA_1c_ of −0.26% in the placebo group was larger, however, than that seen in the previous study of saxagliptin as monotherapy (+0.19%) [3]. Per study protocol, patients received exercise and dietary instruction throughout the study. The reduction in HbA_1c_ observed in the placebo group, and the reduction in body weight in all groups, largest in placebo (−1.3 kg [0.40]), supports the notion that the dietary and exercise interventions (as per local guidelines) were followed by patients in this group. Adherence to diet and exercise interventions in the context of low HbA_1c_ and FPG (at baseline 7.9% and 162 mg/dL, respectively) may have also lessened the placebo-subtracted HbA_1c_ reduction observed, particularly given the glucose-dependent mechanism of action of DPP-4 inhibitors [10].

The reduction in HbA_1c_ from baseline to week 24 observed in all treatment groups was sustained through week 76 in the saxagliptin 2.5 mg q.A.M. and 2.5/5 mg q.A.M. treatment groups. The saxagliptin 5 mg q.A.M. and 5 mg q.P.M. groups, which maintained a higher proportion of patients who completed without titration (17.9%–26.4%) than the saxagliptin 2.5 mg groups (9.3%–13.7%), showed reductions from baseline HbA_1c_ at week 76 that were smaller than those at week 24. The control group, which started metformin treatment at week 24, showed an expected HbA_1c_ reduction from week 24 to week 30 likely due to the addition of active medication but this effect attenuated thereafter. The small number of patients per treatment group and the decreasing amount of available data prior to rescue over time limits the ability to draw definitive conclusions about efficacy results at the end of the long-term period. The reason for the upward HbA_1c_ trend in the arms randomized to saxagliptin 5 mg or placebo is not clear; we can only speculate that it may represent attenuation of the diet and exercise effect documented in the placebo arm during the first 24 weeks and/or natural progression of type 2 diabetes [11]. The fact this trend was not obvious in the 2 arms randomized to 2.5 mg may represent the small effect of titration to 5 mg (Additional file 1).

The 2010 European Medicines Agency draft guidance for drugs for the treatment of diabetes recommends that new therapies be studied in a titration design [12]. In this study, saxagliptin was given as a fixed dose as well as in titration in the first 24 weeks, with all patients eligible for titration in the long-term extension. Decreases in HbA_1c_ were seen in all saxagliptin treatment groups at both the 2.5 and 5 mg doses, and regardless of whether saxagliptin was given in the morning or evening or in a titrated fashion. A small, additional HbA_1c_ reduction was identified with titration of saxagliptin from 2.5 to 5 mg; however, no additional HbA_1c_ reductions were observed with titration of saxagliptin from 5 to 10 mg in the long-term extension. This finding is consistent with other studies, which demonstrated similar efficacy for saxagliptin doses of 5 and 10 mg [4,13]. Dosing saxagliptin 5 mg in the morning resulted in the highest rates of completion and completion without rescue of any treatment group, factors which are likely to be most attractive for many physicians. Importantly, treatment with saxagliptin 2.5 mg with eligibility for early titration to 5 mg did not appear to confer any benefit on safety or tolerability. We found that saxagliptin is generally well tolerated and effective given in titration, but this study does not suggest any advantages to early titration over an initial 5 mg fixed dose.

This study also examined dosing of saxagliptin at different times of the day. The treatment groups initially given saxagliptin 5 mg in the evening showed a decrease from baseline HbA_1c_ that was similar to that in the group initially treated with saxagliptin 5 mg q.A.M., by repeated-measures analysis at weeks 24 and 76. While the FPG results with evening dosing did not reach statistical significance, the magnitude of effect was generally similar to those in the other active treatment groups, and the lack of significance may reflect the relatively small numbers of patients per treatment group. Overall, patients in the saxagliptin 5 mg q.P.M. group had a disposition and safety profile very similar to the other saxagliptin treatment groups.

Saxagliptin was well tolerated. While the proportion of patients experiencing AEs was numerically higher in the saxagliptin groups compared with control the rates of SAEs and AEs leading to discontinuation were similar between saxagliptin-treated and control patients. The overall AE profile observed at week 76 was similar to that seen at week 24. It is important to note that the relatively modest numbers of patients within each treatment group likely contributed to some variability in AE incidence rates.

Trials with lower CV risk patients such as the UK Prospective Diabetes Study (UKPDS) [14] and the Kumamoto study [15], and those with higher risk patients such as the Action to Control Cardiovascular Risk in Diabetes (ACCORD) [16] and Action in Diabetes and Vascular Disease: Preterax and Diamicron Modified Release Controlled Evaluation (ADVANCE) studies [17], have consistently shown a benefit of lower glucose on microvascular complications. However, the anticipated benefit on macrovascular events based on UKPDS lower risk patients [18] was not observed in these studies enriched in patients at higher CV risk. The American Association of Clinical Endocrinologists/American College of Endocrinology [19], ADA [20], and ADA/European Association for the Study of Diabetes [21] guidelines have recently been updated in response, and each emphasizes that hypoglycemia may be a greater risk than previously appreciated, especially in patients at high CV risk. They also suggest that the risk profile of drugs and glycemic targets may be different for populations at higher CV risk. For saxagliptin, the results of this trial in a population at higher baseline CV risk are consistent with the earlier reported monotherapy trial, in which the patient population had a lower baseline CV risk. The overall saxagliptin registrational program did not suggest that saxagliptin was associated with increased CV risk [9]. A large CV outcome trial to investigate the possible benefit of saxagliptin on CV events is currently ongoing (ClinicalTrials.gov Identifier: NCT01107886).

Study limitations include the relatively modest size of the treatment groups, which introduces a greater risk that apparent differences between groups are due to chance. Use of rescue therapy ensured that patients received needed glycemic control. However, it complicates the interpretation of efficacy findings. To avoid the confounding effects of rescue therapy efficacy results included only data collected up to the time of rescue, such that fewer patients were able to contribute data for efficacy analyses at later time points in the study. Because of the small number of patients with available efficacy data at later time points, the long-term efficacy results should be interpreted with caution. The long-term extension control group included patients who received blinded metformin to ensure that patients in the study did not experience overly prolonged periods without antihyperglycemic medication. While necessary for patient well-being, this addition limits the ability to draw firm, placebo-based conclusions with respect to efficacy in the long-term. Finally, there was reduced ability to draw conclusions regarding the PPG-AUC analysis, due to an error in OGTT administration to several participants.

## Conclusions

In conclusion, saxagliptin monotherapy demonstrated HbA_1c_ reductions at 24 weeks, regardless of time of day of administration, and was generally well tolerated for up to 76 weeks, with a low incidence of hypoglycemia across treatment groups.

## Competing interests

Dr. McNeill declares that he has no competing interests. Drs. Frederich, Fleming, and Chen are employees of Bristol-Myers Squibb. Dr. Berglind was an employee of Bristol-Myers Squibb when this work was conducted and is currently an employee of AstraZeneca.

## Authors’ contributions

All of the study authors contributed to the study design, data analysis and interpretation, and/or drafted the manuscript. All authors critically revised and approved the final manuscript.

## Funding

Funding for this study was provided by Bristol-Myers Squibb and AstraZeneca.

## Supplementary Material

Additional file 1 **HbA_1c_ changes from time of first titration in the long-term extension to 13 weeks after time of titration.** Click here for file

Additional file 2 Table summarizing adverse events occurring in ≥ 5% in any treatment group during the short-term period and long-term extension. Click here for file
