# Supplementary material for: The efficacy and safety of the dipeptidyl peptidase-4 inhibitor saxagliptin in treatment-naïve patients with type 2 diabetes mellitus: a randomized controlled trial
Source: Diabetol Metab Syndr. 2012 Jul 24;4:36. doi: 10.1186/1758-5996-4-36 (PMC3541110; doi:10.1186/1758-5996-4-36)
Supplement: Additional file 1 — HbA1c changes from time of first titration in the long-term extension to 13 weeks after time of titration. [file 1758-5996-4-36-S1.doc]

**Additional file 1. HbA_1c_ changes from time of first titration in the long-term extension to 13 weeks after time of titration**

| HbA_1c_ (%) | SAXA 2.5 mg q.A.M.  (n = 54) | SAXA 5 mg  q.A.M.  (n = 56) | SAXA 2.5/5 mg  q.A.M.  (n = 51) | SAXA 5 mg q.P.M.  (n = 53) |
| --- | --- | --- | --- | --- |
| n | 34 | 42 | 27 | 32 |
| Mean at time of first titration (SE) | 7.06 (0.106) | 7.10 (0.084) | 7.01 (0.116) | 7.27 (0.099) |
| Mean at 13 weeks post first titration* (SE) | 6.93 (0.103) | 7.20 (0.101) | 7.13 (0.129) | 7.28 (0.140) |
| Mean change (SE) | –0.12 (0.072) | 0.09 (0.105) | 0.11 (0.086) | 0.02 (0.120) |
| 95% CI | –0.27, 0.02 | –0.12, 0.30 | –0.07, 0.29 | –0.23, 0.26 |

By 13 weeks post-titration, most patients in the saxagliptin 5 mg q.A.M., 2.5/5 mg q.A.M., and 5 mg q.P.M. treatment groups had been titrated to the 10-mg dose, while most of the patients in the 2.5-mg q.A.M. treatment group had been titrated to the 5-mg dose. In this table, the data set of short-term completers was analyzed. *Measurement closest to 13 weeks after time of first titration in the long-term extension period within a window of 10–23 weeks.

SAXA = saxagliptin; SE = standard error.
