# Supplementary material for: The efficacy and safety of the dipeptidyl peptidase-4 inhibitor saxagliptin in treatment-naïve patients with type 2 diabetes mellitus: a randomized controlled trial
Source: Diabetol Metab Syndr. 2012 Jul 24;4:36. doi: 10.1186/1758-5996-4-36 (PMC3541110; doi:10.1186/1758-5996-4-36)
Supplement: Additional file 2 — Table summarizing adverse events occurring in ≥ 5% in any treatment group during the short-term period and long-term extension. [file 1758-5996-4-36-S2.doc]

**Additional file 2.** **Adverse events occurring ≥ 5% in any treatment group: short-term period and long-term extension**

| **Adverse events ≥ 5%, n (%)** | **SAXA 2.5 mg q.A.M.**  **(n = 74)** | **SAXA 5 mg q.A.M.**  **(n = 74)** | **SAXA 2.5/5 mg q.A.M.**  **(n = 71)** | **SAXA 5 mg q.P.M.**  **(n = 72)** | **Control**  **(n = 74)** |
| --- | --- | --- | --- | --- | --- |
| Preferred term |  |  |  |  |  |
| URTI | 11 (14.9) | 10 (13.5) | 11 (15.5) | 11 (15.3) | 7 (9.5) |
| Nasopharyngitis | 3 (4.1) | 4 (5.4) | 3 (4.2) | 5 (6.9) | 3 (4.1) |
| Back pain | 3 (4.1) | 5 (6.8) | 4 (5.6) | 2 (2.8) | 3 (4.1) |
| Diarrhea | 7 (9.5) | 4 (5.4) | 1 (1.4) | 4 (5.6) | 1 (1.4) |
| Headache | 4 (5.4) | 5 (6.8) | 2 (2.8) | 2 (2.8) | 3 (4.1) |
| Pain in extremity | 2 (2.7) | 3 (4.1) | 5 (7.0) | 5 (6.9) | 1 (1.4) |
| Sinusitis | 5 (6.8) | 3 (4.1) | 5 (7.0) | 0 (0.0) | 3 (4.1) |
| UTI | 4 (5.4) | 3 (4.1) | 3 (4.2) | 4 (5.6) | 2 (2.7) |
| Arthralgia | 2 (2.7) | 1 (1.4) | 4 (5.6) | 5 (6.9) | 3 (4.1) |
| Cough | 3 (4.1) | 4 (5.4) | 3 (4.2) | 2 (2.8) | 2 (2.7) |
| Nausea | 4 (5.4) | 2 (2.7) | 4 (5.6) | 3 (4.2) | 1 (1.4) |
| Peripheral edema | 5 (6.8) | 2 (2.7) | 2 (2.8) | 1 (1.4) | 3 (4.1) |
| Hypertension | 3 (4.1) | 3 (4.1) | 4 (5.6) | 0 (0.0) | 1 (1.4) |
| Asthenia | 0 (0.0) | 1 (1.4) | 4 (5.6) | 2 (2.8) | 3 (4.1) |
| Gastroenteritis | 1 (1.4) | 5 (6.8) | 3 (4.2) | 1 (1.4) | 0 (0.0) |
| Dyspepsia | 3 (4.1) | 2 (2.7) | 4 (5.6) | 0 (0.0) | 0 (0.0) |
| Rhinitis | 0 (0.0) | 1 (1.4) | 4 (5.6) | 0 (0.0) | 1 (1.4) |
| Joint injury | 0 (0.0) | 4 (5.4) | 0 (0.0) | 1 (1.4) | 0 (0.0) |

AE: adverse event; SAXA: saxagliptin; URTI: upper respiratory tract infection; UTI: urinary tract infection.
